# COVID-19 Vaccination Reactions and Risk of Breakthrough Infections Among People With Diabetes: Cohort Study Derived From Community Reporters

**DOI:** 10.2196/45536

**Published:** 2024-02-27

**Authors:** Nancy A Dreyer, Kendall B Knuth, Yiqiong Xie, Matthew W Reynolds, Christina D Mack

**Affiliations:** 1 Dreyer Strategies LLC Newton, MA United States; 2 Real World Solutions IQVIA Durham, NC United States; 3 Flatiron New York, NY United States

**Keywords:** COVID-19, diabetes, vaccine, vaccine hesitancy, registry, person-generated health data, patient-reported outcomes, side effects, vaccination, infection, nondiabetic adult, clinical data, fatigue, headache, risk, patient data, medication, community health

## Abstract

**Background:**

This exploratory study compares self-reported COVID-19 vaccine side effects and breakthrough infections in people who described themselves as having diabetes with those who did not identify as having diabetes.

**Objective:**

The study uses person-reported data to evaluate differences in the perception of COVID-19 vaccine side effects between adults with diabetes and those who did not report having diabetes.

**Methods:**

This is a retrospective cohort study conducted using data provided online by adults aged 18 years and older residing in the United States. The participants who voluntarily self-enrolled between March 19, 2021, and July 16, 2022, in the IQVIA COVID-19 Active Research Experience project reported clinical and demographic information, COVID-19 vaccination, whether they had experienced any side effects, test-confirmed infections, and consented to linkage with prescription claims. No distinction was made for this study to differentiate prediabetes or type 1 and type 2 diabetes nor to verify reports of positive COVID-19 tests. Person-reported medication use was validated using pharmacy claims and a subset of the linked data was used for a sensitivity analysis of medication effects. Multivariate logistic regression was used to estimate the adjusted odds ratios of vaccine side effects or breakthrough infections by diabetic status, adjusting for age, gender, education, race, ethnicity (Hispanic or Latino), BMI, smoker, receipt of an influenza vaccine, vaccine manufacturer, and all medical conditions. Evaluations of diabetes medication-specific vaccine side effects are illustrated graphically to support the examination of the magnitude of side effect differences for various medications and combinations of medications used to manage diabetes.

**Results:**

People with diabetes (n=724) reported experiencing fewer side effects within 2 weeks of vaccination for COVID-19 than those without diabetes (n=6417; mean 2.7, SD 2.0 vs mean 3.1, SD 2.0). The adjusted risk of having a specific side effect or any side effect was lower among those with diabetes, with significant reductions in fatigue and headache but no differences in breakthrough infections over participants’ maximum follow-up time. Diabetes medication use did not consistently affect the risk of specific side effects, either using self-reported medication use or using only diabetes medications that were confirmed by pharmacy health insurance claims for people who also reported having diabetes.

**Conclusions:**

People with diabetes reported fewer vaccine side effects than participants not reporting having diabetes, with a similar risk of breakthrough infection.

**Trial Registration:**

ClinicalTrials.gov NCT04368065; https://clinicaltrials.gov/study/NCT04368065

## Introduction

Recent real-world evidence has demonstrated the overall safety and low risk of serious side effects due to COVID-19 vaccines in the general population including using information from community reporters [1]. People with diabetes are of special interest due to their higher risk of hospitalization and death from COVID-19 [2-5]. Here we use a community-based registry in the United States to describe participant-reported data on COVID-19 vaccine side effects and breakthrough infections in people with diabetes and examine whether diabetes medicine use affects the risk of developing vaccine side effects. As a sensitivity analysis of the accuracy of self-reported medication information, we linked data from these registry participants with their health insurance claims for prescription medications to assess the variation of side effects for those who are known to have filled prescriptions for their self-reported diabetes medicines.

## Methods

### Study Design

This is a retrospective cohort study conducted using data provided by community-based adults aged 18 years and older who resided in the United States. The IQVIA COVID-19 Active Research Experience (CARE), an online registry, was created as an observational study of people’s experience with COVID-19 outside of the hospital setting. The initial study purpose was a 1-time survey, launched on April 2, 2020, to capture COVID-19 exposure, medical history, symptoms, and treatments with the goal of identifying any modifiable events that might reduce the severity of infection with COVID-19, such as the use of a dietary supplement, nonprescription medicine, and so forth. It was quickly expanded to include 3 months of follow-up to evaluate symptom persistence. The protocol has been revised 9 times since its launch, including updates as vaccines and boosters were launched, extending follow-up to 12 months, augmenting the symptom list as new information became available, and streamlining to minimize respondent burden. The most recent version of the questionnaire is available online [6,7]. The enrollment was closed in February 2023 [1,8].

The participants were recruited to CARE via periodic outreach through email and social media (Google, Facebook, and Reddit). For this analytic cohort, we selected respondents who received a COVID-19 vaccine and were not part of a COVID-19 vaccine clinical trial. To enroll, participants provided informed consent online, including consent for their data to be matched with pharmacy claims data using a process of deidentification through a trusted third party. At enrollment and follow-up surveys (weekly after vaccination date for 4 weeks and monthly for months 2-12), participants were asked if they met any of the following criteria: had been exposed to COVID-19, had COVID-19–like symptoms, had tested positive for COVID-19, and whether or not they had sought medical care or been admitted to hospital—either for COVID-19–like symptoms or vaccine side effects—and the dates of any such hospitalizations.

### Data Management

The data were extensively curated to eliminate those who were likely to have been under the age of 18 years, were bots, or were such bad typists that the accuracy of their data could not be assured. These data review was performed by looking for patterns where participants consistently chose the first response option to every question, indicated clinically impossible events (eg, pregnant males and height over 7 feet or under 4 feet), or provided nonsensical answers in the free text for side effects), and so forth. The email addresses of volunteers were verified to further rule out attempts at fraudulent data entry.

Since this was designed as an exploratory study, we used all available curated data from CARE. No formal sample size estimates were calculated. There was no imputation of missing data nor was any artificial intelligence, generative or otherwise, used in this data collection or analysis. The gender shown here reflects participants’ self-assessment, noting that transgender or other identity were included as response options.

### Self-Reported Diabetes and Use of Medications for Diabetes

At enrollment, participants reported their demographics and medical history, including whether they had diabetes (without the differentiation of type 1 and type 2 diabetes or prediabetes) and if so, whether they used any prescription medications to treat their diabetes. Those who indicated that they used prescription medications for diabetes were asked to type in the name of the prescription medication they were using.

People who reported having diabetes were compared with those who did not report having diabetes, with further stratification by the type of diabetes medication used (using the most frequently reported medications, ie, insulin without metformin, insulin and metformin, metformin without insulin, or neither).

The accuracy of self-reported insulin and metformin use was confirmed by comparison with IQVIA Prescription Claims data [9,10], which as of November 2022, included data from roughly 92% of retail pharmacies, 72% of standard mail service, and 76% of long-term care facilities in the United States. Deidentified CARE data were matched with pharmacy claims data (filled within 6 months before or after study enrollment to capture delayed claims and large refill quantities) using the National Drug Code and product name. These linked prescription claims data were used as a sensitivity analysis to examine vaccine side effects for diabetes medications confirmed in pharmacy claims.

### COVID-19, Vaccinations, Side Effects, and Breakthrough Infections

At both enrollment and follow-up surveys, participants were asked to report if they had been tested for COVID-19 and, if so, test dates and results; whether they had been vaccinated against COVID-19; and what prescription and nonprescription medications they used, as well as dietary supplements and complementary medicines [8]. If they reported having been vaccinated against COVID-19, they were asked to report the vaccine manufacturer, date, and lot number. They were also asked if they experienced any side effects after the vaccination and were provided a list of 13 symptoms. They also had the option to insert additional side effects using a free text field for side effects that were not listed.

All CARE participants who reported completion of a COVID-19 vaccine regimen approved by the US Food and Drug Administration (2 doses of Pfizer or Moderna or 1 dose of Johnson & Johnson) between March 19, 2021, when vaccine side effect questions were first added to CARE, and July 16, 2022, were included in this analytic cohort.

### Analysis

No statistical tests were used in these exploratory evaluations of diabetes medication-specific vaccine side effects. Vaccine side effects are described based on the total number reported per participant (means and SDs) and percentages for individual side effects. For 2-dose vaccines, each side effect was counted once regardless of whether it was reported at only 1 dose or at both doses. Side effects entered as free text were manually reviewed and grouped into related categories. The distribution of self-reported vaccine side effects by diabetes medications is illustrated graphically to support the examination of the magnitude of side effect differences for various medications and combinations of medications used to manage diabetes.

Incidences of breakthrough infections are described according to whether respondents reported that they had diabetes at enrollment. Breakthrough infections were defined in alignment with the US Centers for Disease Control and Prevention as a positive COVID-19 test, regardless of the type of test, after 14 days post completion of a vaccine regimen [11].

Multivariate logistic regression was used to estimate the adjusted odds ratios (aORs) of vaccine side effects or breakthrough infections by diabetic status, adjusting for age, gender, education, race, ethnicity (Hispanic or Latino), BMI, smoker, receipt of an influenza vaccine, vaccine manufacturer, and all medical conditions.

### Ethical Considerations

This study was reviewed and approved by an external institutional review board (Advarra; Pro00043030) and registered with ClinicalTrials.gov (NCT04368065) in the spirit of full disclosure, although this was not a clinical trial. This study fully complies with the Declaration of Helsinki.

## Results

### Study Population

**Figure 1:**
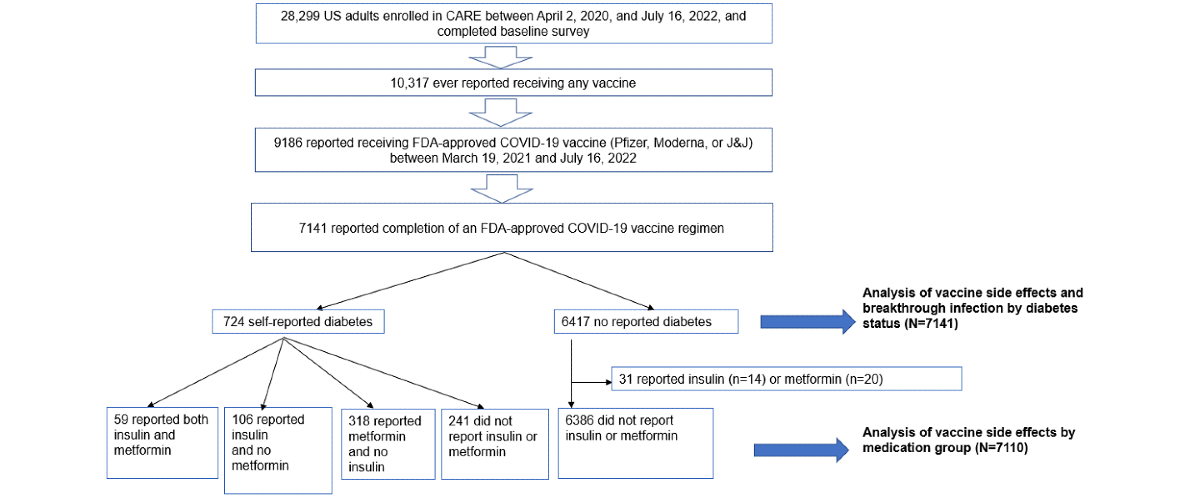
Flowchart of analysis populations from CARE registry. CARE: COVID-19 Active Research Experience; FDA: US Food and Drug Administration.

A flowchart describing the study population is shown in Figure 1. The analysis population was composed of 7141 participants who reported having completed a vaccine regimen between March 19, 2021, and July 16, 2022, with 724 reporting they had diabetes and 6417 participants who did not report so (people without any note of having diabetes). The median follow-up time from completion of a vaccine regimen to the last survey submitted was 170 (IQR 38.0-319.5) days and 145 (IQR 37.0-314.0) days for those with and without diabetes, respectively. Most people with diabetes used insulin (n=165, 22.8%), metformin (n=318, 43.9.1%), or both (n=59, 8.1%).

### COVID-19 Vaccinations and Side Effects Among People With Diabetes

In this study population, people with diabetes reported fewer vaccine side effects than those without diabetes (mean 2.7, SD 2.0 vs mean 3.1, SD 2.0, respectively; Table 1), although respondents with diabetes were older than nondiabetics and reported more comorbidities, including hypertension, obesity, depression, and autoimmune disorders.

**Table 1:** Characteristics at enrollment survey and side effects of COVID-19 vaccines reported by participants at the first or second vaccine dose, by self-reported diabetic status.

		All participants (N=7141)	
		People with diabetes (n=724)	No reported diabetes (n=6417)
**Days of follow-up from completion of COVID-19 vaccine regimen** **(ie, last dose in regimen), n**		724	6417
	Mean (SD)	182.1 (144.4)	175.0 (143.9)
	Median (IQR)	170.0 (38.0-319.5)	145 (37.0-314.0)
	Range	0-598.0	0-747.0
**Age (years), n**		724	6417
	Mean (SD)	57.8 (12.04)	47.5 (15.57)
	Median (IQR)	60 (51.0-66.0)	46 (34.0-61.0)
**Age group (years), n**		724	6417
	18-29, n (%)	14 (1.9)	767 (12.0)
	30-39, n (%)	46 (6.4)	1704 (26.6)
	40-49, n (%)	106 (14.6)	1013 (15.8)
	50-59, n (%)	193 (26.7)	1104 (17.2)
	>60, n (%)	365 (50.4)	1829 (28.5)
**Gender** **, n**		724	6417
	Self-described as female, n (%)	552 (76.2)	5375 (83.8)
**Race, n**		724	6409
	Black, n (%)	19 (2.6)	100 (1.6)
	White, n (%)	651 (89.9)	5793 (90.4)
	Other, n (%)	54 (7.5)	516 (8.1)
**Ethnicity,** **n**		720	6402
	Hispanic or Latino, n (%)	39 (5.4)	369 (5.8)
**BMI, n**		714	6286
	Underweight or normal weight (15.0≤BMI<25.0), n (%)	66 (9.2)	1802 (28.7)
	Overweight (25.0≤BMI<30.0), n (%)	150 (21.0)	1825 (29.0)
	Obese (30.0≤BMI≤40.0), n (%)	323 (45.2)	2026 (32.2)
	Severe obesity (BMI>40.0), n (%)	175 (24.5)	633 (10.1)
**Education, n**		720	6406
	High school or less, n (%)	89 (12.4)	525 (8.2)
	Some college, n (%)	273 (37.9)	1845 (28.8)
	4 year college degree, n (%)	140 (19.4)	1731 (27.0)
	>4 year college degree, n (%)	218 (30.3)	2305 (36.0)
**Smoker, n**		671	6181
	Yes, n (%)	73 (10.9)	571 (9.2)
**Vaccinated for influenza, n**		721	6358
	Yes, n (%)	568 (78.8)	4659 (73.3)
**Other medical conditions, n**		724	6410
	Hypertension, n (%)	409 (56.5)	1286 (20.1)
	Depression, n (%)	294 (40.6)	2007 (31.3)
	Insomnia or trouble sleeping, n (%)	275 (38.0)	1889 (29.5)
	Anxiety, n (%)	272 (37.6)	2515 (39.2)
	Autoimmune disease, n (%)	146 (20.2)	732 (11.4)
	Cardiovascular disease, n (%)	129 (17.8)	311 (4.9)
	Lung disease, n (%)	113 (15.6)	585 (9.1)
	Kidney disease, n (%)	70 (9.7)	176 (2.7)
	Blood disorder, n (%)	36 (5.0)	150 (2.3)
**Manufacturer of COVID-19 vaccine received, n**		724	6417
	Pfizer, n (%)	327 (45.2)	3124 (48.7)
	Moderna, n (%)	317 (43.8)	2502 (39.0)
	J&J, n (%)	80 (11.0)	791 (12.3)
**Categories of number of side effects to COVID-19 vaccines, n**		724	6417
	No side effects, n (%)	93 (12.8)	530 (8.3)
	1 to 2 side effects, n (%)	300 (41.4)	2186 (34.1)
	3 or more side effects, n (%)	331 (45.7)	3701 (57.7)
**Number of side effects to COVID-19 vaccines, n**		724	6417
	Mean (SD)	2.7 (2.0)	3.1 (2.0)
	Median (IQR)	2 (1.0-4.0)	3 (2.0-5.0)
	Range	0-9	0-10
**Specific side effects to COVID-19 vaccines, n**		724	6417
	Injection site reactions, n (%)	530 (73.2)	4995 (77.8)
	Fatigue, n (%)	435 (60.1)	4484 (69.9)
	Headache, n (%)	284 (39.2)	3126 (48.7)
	New or worsening muscle pain, n (%)	177 (24.4)	1928 (30.0)
	Fever, n (%)	171 (23.6)	1938 (30.2)
	New or worsening joint pain, n (%)	142 (19.6)	1360 (21.2)
	Nausea or vomiting, n (%)	91 (12.6)	1016 (15.8)
	Swollen lymph nodes, n (%)	71 (9.8)	724 (11.3)
	Chills, n (%)	20 (2.8)	312 (4.9)
	Diarrhea^a^, n (%)	<10	72 (1.1)
	Dizziness^a^, n (%)	<10	76 (1.2)
	Severe allergic reaction^a^, n (%)	<10	35 (0.5)

^a^Percentage not shown for <10 responses.

**Figure 2:**
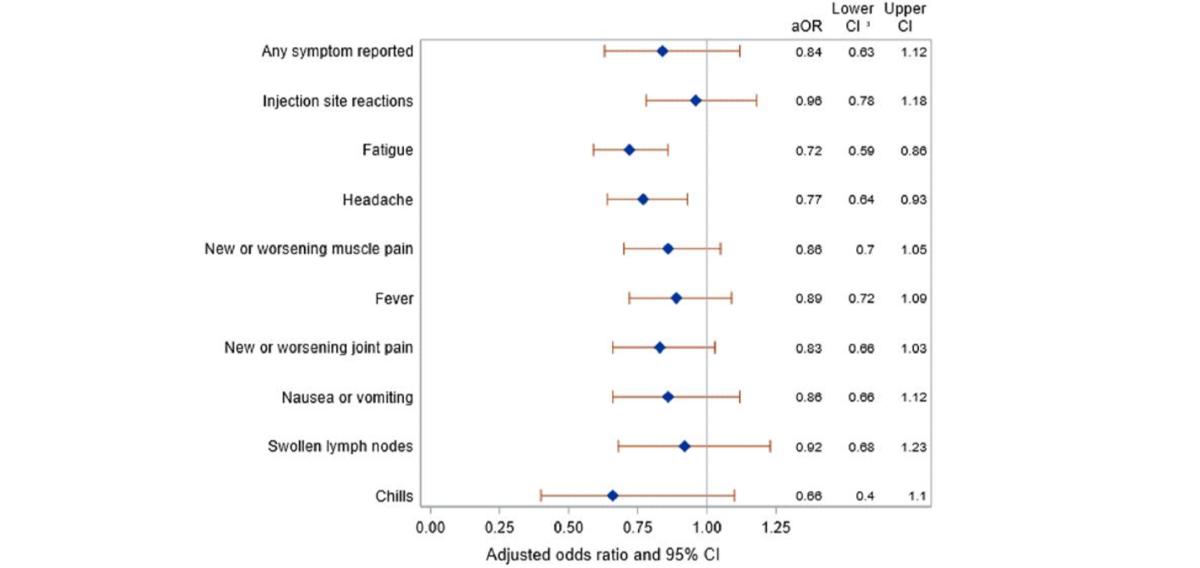
Adjusted (adjusted for age, gender, education, race, ethnicity, BMI categories, smoking status, receipt of an influenza vaccine, vaccine manufacturer, and all medical conditions) odds ratios comparing COVID-19 vaccine side effects (diarrhea, dizziness, and severe allergic reaction not reported due to small numbers) between people with diabetes (n=724) and without diabetes (reference group, n=6417). aOR: adjusted odds ratio.

**Figure 3:**
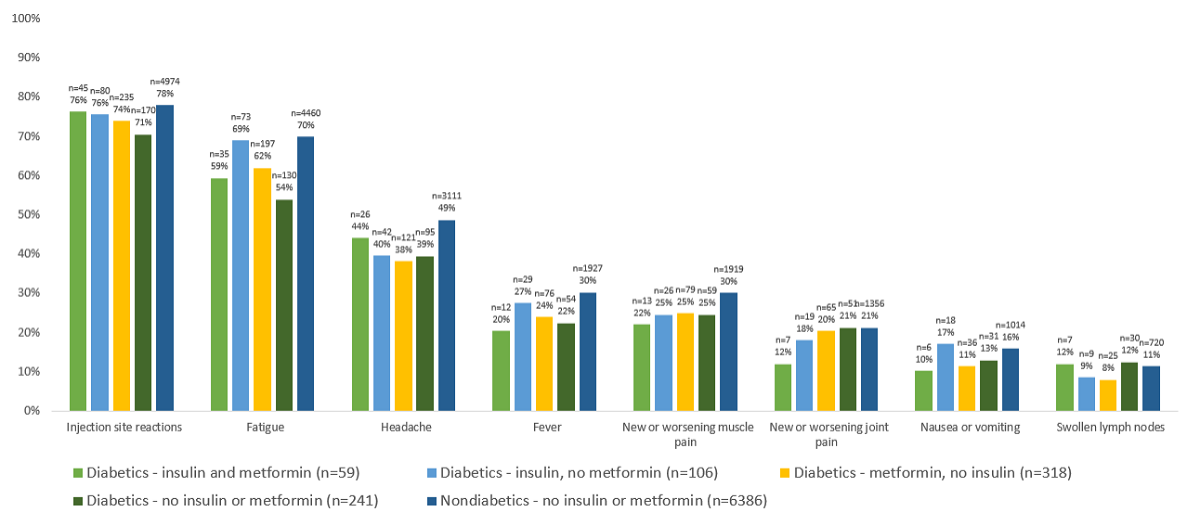
COVID-19 vaccine side effects comparing self-reported diabetes medication use among diabetes to those without diabetes (n=7110). Note that 31 people were excluded here who did not report having diabetes but who did report using insulin or metformin for treatment of another medication condition.

**Figure 4:**
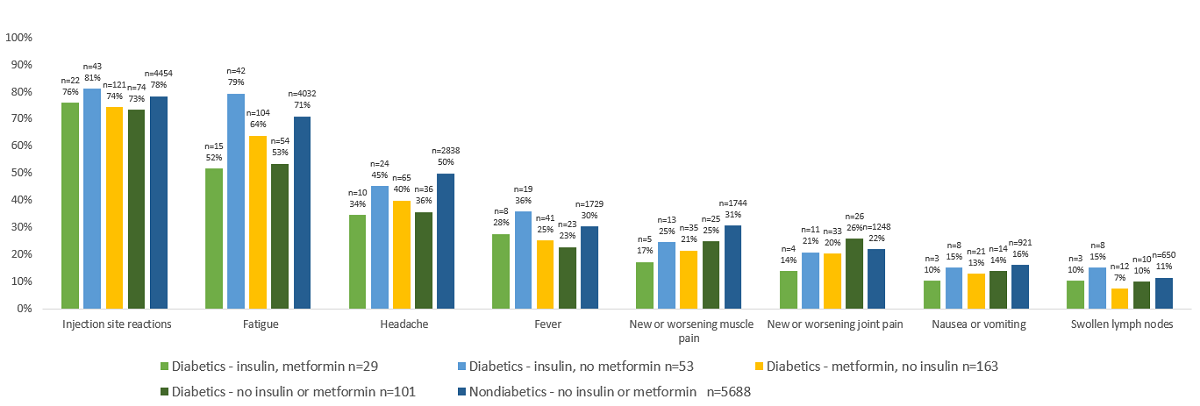
COVID-19 vaccine side effects by diabetes and diabetes medications confirmed through linked pharmacy claims (n=5034).

The aORs for having any or individual vaccine side effects were consistently lower for participants reporting having diabetes compared with those not reporting diabetes, with notable reductions in the risk of side effects such as fatigue and headache (Figure 2). Specific diabetes medications affected the risk of various side effects (Figures 3 and 4), but no consistent patterns of risks were observed between medications or side effects. A similar pattern of vaccine side effects by diabetes medication use was observed in a sensitivity analysis restricted to diabetes drugs that were confirmed in prescription claims (Figure 4).

### Accuracy of Self-Reported Medication Use

Most self-reported diabetes medication use was confirmed in prescription claims for participants in the analysis population, who indicated using prescription medications and were linked to pharmacy claims within 6months before or after enrollment in CARE. Specifically, among 142 participants with diabetes who reported using insulin in CARE, 101 had linked prescription claims data available for analysis; using these linked data, 81.2% (82/101) showed at least 1 claim for insulin. Of the 325 participants reporting diabetes who reported using metformin in CARE, 228 had linked prescription claims data and 84.2% (192/228) showed at least 1 claim for metformin.

### Breakthrough Infections After Vaccination

Breakthrough infections through participants’ last survey were reported by 36 (5.0%) participants reporting diabetes and 396 (6.2%) participants not reporting having diabetes. The median time to breakthrough infection for those who were fully vaccinated was similar between participants reporting diabetes (252, IQR 139-280 days) and participants not reporting diabetes (265, IQR 200-317 days; *P*=.10). When adjusting for other factors, there was no meaningful difference in the risk of breakthrough infections between participants reporting and not reporting diabetes (aOR 0.95, 95% CI 0.65-1.40).

## Discussion

### Principal Findings

This observational study showed that participants reporting diabetes experienced a lower risk of vaccine side effects than participants not reporting diabetes, even when higher BMI, more frequent comorbidities, and other differential risk factors were controlled statistically. This is similar to findings from another digital real-world study by Beatty et al [12] that showed the presence of self-reported diabetes was not associated with increased risk of COVID-19 vaccine side effects, despite some difference in the time frame of side effects measurement (ie, 2 weeks in CARE vs monthly reporting by Beatty et al [12]).

In general, those who used diabetes medications reported fewer side effects than those who did not report having diabetes or used metformin for any purpose. The most notable exception was evident in the incidence of fatigue; here participants who used insulin reported having levels of fatigue higher than (Figure 4) or equal to (Figure 3) those without diabetes. Analysis of only those medications confirmed by prescriptions also showed slightly higher rates of fever, swollen lymph nodes, and injection site reactions among insulin users compared to those who did not report having diabetes or using metformin, though it is important to emphasize that these are small differences derived from the analysis of relatively small numbers.

The reasonably high correlations between self-reported insulin and metformin with pharmacy claims (81.2%, 82/101 and 84.2%, 192/228, respectively) were similar to findings from other comparisons of adult self-reported prescription data and national pharmacy claims data, noting that even using a national prescription registry in this earlier work, which was presumed to have 100% coverage of the population, did not show 100% agreement with self-reported medication use [13].

### Comparison to Prior Work

This level of agreement between self-reported prescription medication use and pharmacy health insurance claims for those medications not only lends more weight to the findings derived from self-reported data but also reinforces the value of participant-reported health data [14].

Some literature shows that people with diabetes have lower neutralizing antibodies after receiving COVID-19 vaccines than the general population [15,16], raising the question of whether people with diabetes are adequately protected by vaccination. However, this study confirms the work of Beatty et al [12] and adds information on breakthrough infections, showing that participants reporting diabetes did not experience any higher rates of breakthrough infections than their counterparts not reporting diabetes, regardless of side effects after vaccination for COVID-19.

### Strengths and Limitations

#### Strengths

This study was designed as an exploratory study of COVID-19 in the community setting, including the risks and benefits of vaccination. Its main strength is bringing the voice of the people to the forefront, without any interpretation or editing by medical care providers.

#### Limitations

First, voluntary participation in online surveys is susceptible to bias. A fundamental assumption used here is that volunteers will answer honestly, especially since there was no remuneration or other benefit for participation. This study builds on work conducted previously [14] using this methodology where participants from Denmark self-reported prescription medication use was validated through a national prescription registry, with similar levels of reporting agreement shown here. Further, this study also confirms that valuable information can be obtained from laypeople, including information that may not otherwise be available such as perception of vaccine-related side effects. In this study, there was no clinical validation of self-reported side effects nor was proof of test-confirmed COVID-19 requested. These decisions were made to minimize participant burden and to support full reporting of participants’ experience about how they felt after vaccination for COVID-19, that is, whether or not they sought medical care. The perception of side effects is important, regardless of how they are viewed by a clinician since they shape personal behavior [17].

Second, we did not differentiate between prediabetes, type 1 and type 2 diabetes, largely since this was a general survey of laymen and we were concerned that not all people would be able to respond accurately. Nor did we seek information about glucose levels due to the broad nature of this study. Instead, we attempted to strengthen our conclusions by analyzing vaccine side effects according to the use of diabetes medications only among those respondents who also indicated that they had diabetes and excluding people from our analysis of diabetes medications who reported using metformin and insulin but did not report having diabetes.

Third, generalizability and missing data are concerns for every observational study. Despite participation from all 50 states, adults who join CARE are not representative of the US population in general or all people with diabetes. The CARE participants are more highly educated than the general population as is common in online research [12,14]. Most described themselves as Caucasian females, aged 30-50 years, and the responses of these unpaid volunteers reflect the experience of people who had both the time and interest to respond to internet advertisements on social media. That said, comparisons within this study population are unlikely to be subject to selection biases that would cause differential reporting between participants reporting or not reporting diabetes. Furthermore, there was no effort to specifically recruit people with diabetes, nor any advance notice of the intent to study vaccine side effects specifically or to compare side effects according to the medication use. However, people who had severe reactions from COVID-19 vaccination may not have participated in this study or may not have provided follow-up due to hospitalization or death.

Finally, most of these data were collected when the predominant COVID-19 variants were the Delta and the original Omicron (BA.1 and BA.2.12.1) variants. The rates of breakthrough infections may differ for other variants [18].

### Conclusions

Overall, these results should provide assurance that simply having diabetes does not increase the risk of vaccine side effects compared with those not reporting diabetes. In fact, the risk of developing vaccine side effects in participants reporting diabetes appears lower than in those not reporting diabetes, without any increased risk of breakthrough infections after vaccination.

## Abbreviations

aOR: adjusted odds ratio
CARE: COVID-19 Active Research Experience

## Data Availability

Deidentified data sets generated during this study are available from the corresponding author upon reasonable request.
